# Does elite European match-play affect salivary immunoglobulin-A and cortisol in soccer players? The influence of playing status and match outcome

**DOI:** 10.3389/fphys.2024.1253417

**Published:** 2024-01-25

**Authors:** R. Morgans, R. Oliveira, D. Rhodes, P. Orme, H. I. Ceylan, F. T. González-Fernández, A. Linán-González, A. Moreira

**Affiliations:** ^1^ School of Sport and Health Sciences, Cardiff Metropolitan University, Cardiff, United Kingdom; ^2^ Research Centre in Sports Sciences, Health and Human Development, Vila Real, Portugal; ^3^ Sports Science School of Rio Maior–Polytechnic Institute of Santarém, Rio Maior, Portugal; ^4^ Football Performance Hub, Institute of Coaching and Performance, School of Sport and Health Sciences, University of Central Lancashire, Preston, United Kingdom; ^5^ Sport Science and Medical Department, Bristol, United Kingdom; ^6^ Faculty of Kazim Karabekir Education, Physical Education of Sports Teaching Department, Ataturk University, Erzurum, Türkiye; ^7^ Department of Physical Education and Sports, Faculty of Sport Sciences, University of Granada, Granada, Spain; ^8^ Department of Nursing, Faculty of Health Sciences, Melilla Campus, University of Granada, Melilla, Spain; ^9^ Department of Sport, School of Physical Education and Sport, University of Sao Paulo, São Paulo, Brazil

**Keywords:** football, immune function, hormonal response, recovery, starting status, soccer

## Abstract

**Introduction:** The aims of this study were to: a) investigate salivary immunoglobulin A (s-IgA) and cortisol (s-Cort) responses to nine competitive fixtures in starting and non- starting soccer players; and b) compare s-IgA and s-Cort responses of starters and non-starters considering match outcome.

**Methods:** Saliva from 19 male outfield players from an elite soccer team (mean ± SD, age 26 ± 4 years; weight 80.5 ± 8.1 kg; height 1.83 ± 0.07 m; body-fat 10.8% ± 0.7%) was collected. Saliva samples were taken on the day before each match (MD-1), 60-min before kick-off (MDpre), 30-min post-match (MDpost), and 72-h post-match (MD+3). There were five wins, one draw and three losses.

**Results:** The mean s-IgA value was found to be significantly lower at MD+3 compared to MDpre and MDpost. s-Cort was significantly higher at MDpost compared to MD-1 and MDpre. When compared to MDpre, a statistically significant decrease in s-Cort was observed at MD+3 compared to MDpost. Starters displayed higher s-Cort values across the nine matches. There was a significant group-by-time interaction for s-Cort. There was a significant increase in s-Cort levels at MDpost compared to MD-1 and from MDpre to MDpost in starting players. At MDpost, starters had significantly higher s-Cort values. s-IgA values of starting and non- starting players following successful and unsuccessful matches did not reveal a significant difference. However, similar analysis of s-Cort in successful matches showed a significant difference between starters and non-starters. s-IgA values at MD-1, MDpre, MDpost and MD+3 in starters and non-starters following successful and unsuccessful matches revealed significant differences at MDpre and MDpost in starters, respectively. Furthermore, s-Cort values at MD-1, MDpre, MDpost and MD+3 in starters and non-starters in successful and unsuccessful matches revealed significant differences at MD+3 in starting players.

**Discussion:** The present study suggests that in elite level soccer players, both starting status and match outcome influence s-IgA and s-Cort responses, particularly starters. Specifically, s-IgA was lower for starters before and after the match following successful outcomes. Moreover, higher s-Cort values were found before the match while lower values occurred after the match for starters in successful matches.

## Introduction

The physiological demands of soccer performance and the resulting impact on the body are relatively well understood (Nedelec et al., 2012; Nedelec et al., 2013; Bangsbo, 2014). More recent research has shifted in an attempt to understand the varying demands that may be experienced by different players within the same squad as a result of their individual involvement in matches and training sessions (Anderson et al., 2016). It is well known that while some players may be consistently selected to play matches, there are also players whose role within the team is less significant and subsequently limits match-play exposure. Practically, this involves grouping players into a “starters” group which represents players who play the majority of match minutes available throughout the season, and “non-starters” who play considerably less minutes throughout the season. Anderson et al. (2016) suggest that “non-starters” are players who play less than 30% of all available match minutes over the course of a season, while other studies suggested that non-starting players completed less than 60-min in at least three consecutive matches (Martin-Garcia et al., 2020; Oliveira et al., 2020; Oliveira et al., 2021).

Various methods have been used within research in an attempt to quantify the physiological impact following soccer match-play (Nedelec et al., 2012; Nedelec et al., 2013). As outlined earlier, Morgans et al. (2022) stated that understanding individual changes in the physiological status of soccer players may allow practitioners to individualize player schedules and programmes to optimize physical preparation and maximize future performances. Furthermore, the importance of using methods such as salivary sampling to quantify markers including immunoglobulin A (s-IgA) (Morgans et al., 2014a; Morgans et al., 2014b; Rico-Gonzalez et al., 2021; Rico-Gonzalez et al., 2014) and cortisol (s-Cort) (Moreira et al., 2009) in order to understand respectively, mucosal immune and hormonal responses of players following match-play have been outlined. This in turn may also provide a better understanding of a player’s susceptibility to illness during a period of exercise induced immune system suppression (Nakamura et al., 2006; Mortatti et al., 2012) and its inherent interplay with individual responses to stress.

In a recent review, Springham et al. (2022) emphasized that biomarkers related to player “stress balance,” immunological factors (such as s-IgA), and hormonal indicators are now commonly utilized in soccer. The authors highlighted evidence demonstrating the well-established association between s-IgA and training or match load, highlighting the sensitivity to changes in psychophysiological stress and the heightened risk of compromised mucosal immunity. This relationship is further supported by the close connection between s-IgA levels and symptoms of upper respiratory tract infections (URTI), as well as its correlation with perceived fatigue in soccer players. Based on the insights from this review, Springham et al. (2022) argue that there is ample evidence to support the inclusion of s-IgA and cortisol measurements as part of a multivariate, individualized player monitoring system in professional soccer.

However, despite this evidence, Morgans et al. (2014b) had earlier presented data that showed fluctuations in s-IgA to be sensitive to changes in the physical demands placed on soccer players as a result of changes in fixture scheduling at different time points across the season. Values for s-IgA decreased during periods of condensed fixture schedules (2-3 matches per week) but returned to “normal” baseline measures when the regular fixture schedule resumed (one match per week). Additionally, a recent systematic review examining training load and s-IgA in soccer suggested this variable as a valid measure to control the mucosal immune function of players, and that the impact of non-official matches and periods with a single match per week in s-IgA is not clear (Rico-Gonzalez et al., 2021), which suggests more research is warranted. Furthermore, another systematic review of s-IgA in other sports also suggested that lower s-IgA values occurred following greater training load (intensity/volume) and during congested periods. Thus, the management of such variables has been recommended (Rico-Gonzalez et al., 2021b). Indeed, the influence of other situational (contextual) factors was previously demonstrated in soccer. Freitas et al. (2016) examined s-IgA responses to official and simulated competitive matches in young soccer players and demonstrated a significant decrease in s-IgA (absolute concentration and rate of s-IgA secretion) for official matches from pre-to post-match. The findings of this study suggest that official matches may lead to a decrease in the main mucosal immunity function parameter in soccer players, increasing the risk of URTI. Moreover, this finding also suggests that additional factors such as match intensity, competitive anxiety, and match outcome may impose different levels of stress on team sport players, leading to varying responses in mucosal immunity.

Furthermore, concerning the stress response, it is important to mention that psychological stress is believed to influence mucosal immune function. Prolonged periods of psychological stress have been linked to a reduction in s-IgA concentrations (Deinzer et al., 2000). More recently, stress has been redefined as “the individual state of uncertainty about what needs to be done to safeguard physical, mental, or social wellbeing” (Peters et al., 2017). In such cases, uncertainty (entropy) monitored by the anterior cingulate cortex stimulates the amygdala, increasing Locus coeruleus, sympathetic nervous system, and hypothalamic-pituitary-adrenal axes activity, facilitating the search for a novel strategy. Due to the association between stress and its mechanisms with mucosal immune function (Bishop and Gleeson, 2009), it is relevant to assume that different match outcomes and the manner that players cope with stress may imply varying mucosal immune function responses, which, in turn, can be monitored by salivary cortisol (s-Cort) and s-IgA concentration, considering match result.

In this sense, while on the other hand, Moreira et al. (2009) compared s-Cort values before and after a competitive training match in two top-level male professional soccer teams. However, no significant changes were found between either teams’ pre-versus post-match. Additionally, another study (Morgans et al., 2022) found no significant difference between match-day minus one (MD-1) and 60-min before the match kick-off (MDpre). The same study observed a significant increase from 60-min before kick-off to 30-min post-match, while at 48-h post-match, s-Cort showed a decrease though still slightly higher than 60-min before kick-off, with no significant differences. Contrasting to the previous results, a study examining professional female soccer players found that s-Cort increased following two matches, although it is relevant to note that the matches were competition finals (Maya et al., 2016).

Nonetheless, an important limitation of the previous studies (Moreira et al., 2009; Maya et al., 2016) may be the pooling of players irrespective of starting status (i.e., “starters” vs. “non-starters”). The findings of this type of research may be considerably different if assessed in relation to the starting status of players. For instance, higher values for non-starting players were observed during training sessions completed on the day following match-day for total distance, high-speed running and sprint distance (Martín-Garcia et al., 2018). Additionally, higher accumulated weekly loads of external load measures (e.g., total distance and sprint distance) were found for starters than non-starters (Gholizadeh et al., 2022).

Furthermore, none of the previous research considered the contextual variable of match outcome which may influence the immunological and hormonal recovery profiles of players (Morgans et al., 2022). Although, previous research revealed that winning matches produced higher external load than a draw or loss (Reche-Soto et al., 2019) thus, consequently, will affect the subsequent training and competition. Moreover, a previous study analysed if training load varied considering the next outcome of the match and showed a tendency of lower high-speed running distance (between MD-5 to MD-1) when the next match was a win than when it was a loss (Oliveira et al., 2020). However, no s-IgA and s-Cort responses was considered which may have additional implication for coaches. Thus, a greater understanding of how this situational variable might affect salivary biomarker measures may help coaches and practitioners to ensure more informed decisions are made when prescribing subsequent training load and recovery.

Therefore, the aim of this study was to investigate s-IgA and s-Cort responses to nine competitive fixtures compared with baseline and pre-match values during the second phase of a competitive season (season 2021–22) in soccer players classified as starters (n = 10 ≥ 60% of matches) and non-starters (n = 9 < 60% of matches). Furthermore, the study aims to determine any differences in the objective s-IgA and s-Cort markers following the post-match recovery period for starters and non-starters. This study re-analyses s-IgA, s-Cort and match performance data from previously published work (Morgans et al., 2022). However, in addition, this study also aims to compare s-IgA and s-Cort responses of starters and non-starters considering the match outcome (matches won *versus* matches drawn or lost). Based on previous literature (Morgans et al., 2014b; Freitas et al., 2016; Reche-Soto et al., 2019; Morgans et al., 2022), the study hypothesis was that differing s-IgA and s-Cort responses may be observed when considering the starting status of elite soccer players and the match outcome (win, draw, loss).

## Materials and methods

### Participants

The current investigation collated data from a cohort of 19 male outfield players from an elite professional soccer team (mean ± SD, age 26 ± 4 years; weight 80.5 ± 8.1 kg; height 1.83 ± 0.07 m; body-fat 10.8% ± 0.7%). The participants had been playing soccer for a minimum of 8 years. The inclusion criteria for the study included: i) listed on the roster of the first-team squad of the Premier League club at the start of the 2021–22 season, ii) regularly competed in the first-team, iii) participated in ≥60% of matches, iv) completed 45-min playing time in two (20%) or more league matches v) did not use dietary supplements during the study, vi) were un-injured over the course of the study, and vii) did not participate in another training program along with this study. Additionally, the exclusion criteria for the study included: i) long-term injury, ii) player joining the team late in either of the study seasons, iii) lack of full, complete match-play data, iv) and goalkeepers, due to the different variations in the physical demands with outfield players. Notably, this study re-analyses the same data collected from the sample cohort recently examined (Morgans et al., 2022).

The sample were initially recruited based on squad selection across nine league matches, home matches (n = 5), away fixtures (n = 4) in which five were successful (win) and four were unsuccessful (draw or loss) during the 2021–22 season. Only data recorded during home and away official league matches were included in the present study. The sample was further sub-divided into starters (n = 10 ≥ 60% of matches) and non-starters (n = 9 < 60% of matches) (Nobari et al., 2020; Morgans et al., 2022). A minimum sample size of 14 was determined from an *a priori* statistical power analysis using G_Power (Version 3.1, University of Dusseldorf, Germany) (Faul et al., 2009). The power analysis was computed with the following equation was used: *Sample Size* = *Z*2 × (*p*) × (1 − *p*)/*C2*, where Z = confidence level (95%); *p* = 0.05 and *C* = margin of error 0.05.

All data evolved as a result of employment in which players were routinely monitored over the course of the competitive season, nevertheless, approval for the study from the club was obtained (Winter and Maughan, 2009) and written informed consent was obtained from all participants. The study was conducted according to the requirements of the Declaration of Helsinki and ethics was approved by the local Ethics Committee of University of Central Lancashire (N 0104 dated 7/12/20). To ensure confidentiality, all data were anonymized before analysis. Participants were fully familiarized with the experimental procedures within this study due to the regular testing protocols implemented as part of the clubs’ performance monitoring strategy. During the study, players were instructed to maintain normal daily food and water intake, and no additional dietary interventions were undertaken.

### Procedures

A quasi-experimental approach with pre- and post-match measures to track stress biomarkers was employed, with data pooled across all matches and analyzed according to starting status and match outcome.

The study period included saliva sampling and all match performance across a 10-week phase during the second phase of an elite European competitive season of the 2021–2022 season. The training sessions performed during the investigation were representative of a typical training micro-cycle implemented within elite European soccer, involving a periodize training week encompassing low, moderate, and high intensity sessions leading to competitive match-play. Only team pitch-based training sessions were included for analysis. All other sessions, individual training sessions, recovery sessions, and rehabilitation training sessions were excluded. All training sessions were integrated to include technical, tactical, physical and mental components. All players completed one or two strength and power gym-based sessions per microcycle incorporating upper and lower body and core exercises, although these sessions were not included in the analyses as mentioned earlier. All running training data was collected at the club’s official training facility.

### Salivary sampling and analysis

For professional soccer players, s-Cort levels pre-match and post-match have previously been reported (Moreira et al., 2009). Thus, players provided saliva samples pre-breakfast (between 09.30 and 10.30 a.m.) approximately 60-min before training on MD-1, 60-min before kick-off on MD (MDpre), 30-min following each match (MDpost) and pre-breakfast approximately 60-min before MD+3 training. Given that soccer match-play induces a reduction in s-IgA levels that return to basal levels within 18-h (Fredericks et al., 2012), it was reasoned that collection of samples immediately post-match (MDpost) and at post-match (MD+3) would allow to a recovery profile of the acute suppression effects on s-IgA levels from that associated with more chronic levels of stress to be ascertained. Except on MD, all participants were in a fasted state and required to abstain from food and caffeine products for a minimum of 2-h prior to the collection of saliva, and all salivary samples were collected at the same time of day (09.30–10.30 a.m.) and under similar environmental conditions (temperature: 19°C–23°C) for all participants to minimize any residual effect of exercise, circadian and atmospheric variations. Match-day sample collection time varied due to the official start of the match but was consistently 60-min prior to kick-off.

The saliva samples were collected and analyzed from this cohort of players using the Soma OFC II collection kits in combination with real-time Lateral Flow Device (LFD), respectively. This method has been previously validated for oral fluid collection in the immunoassay of immunoglobulins in sports persons (MacDonald et al., 2017) and correlates well with other methods (enzyme-linked immunosorbent assay) adopted in the determination of s-IgA (Morgans et al., 2015) and s-Cort (Venkataraman et al., 2007; Moreira et al., 2009; Morgans et al., 2014b). The manufacturer’s guidelines, that have recently been employed in elite soccer players (Morgans et al., 2022) were followed for saliva collection. This method has been previously validated (Dunbar et al., 2011; MacDonald et al., 2017) against ELISA (*r* ^2^ = 0.78) in 208 samples collected from a cohort of English Premier League soccer players (Dunbar et al., 2011). Again, for this analysis, data were taken on MD-1, MDpre, MDpost and MD+3 to ensure comparison of consistent daily data in relation to training and match-play and no training was performed outside the formal team training schedule.

### Statistical analysis

Descriptive statistics were calculated and presented as mean ± standard deviation. Indeed, were shown in scatter plots. First, the normality of numerical variables was evaluated using the Shapiro-Wilk test. The model residuals were found to have a normal distribution (*p* > 0.05). Mauchly’s test determined the sphericity assumption. Sphericity assumption was provided for s-IgA and s-Cort (*p* > 0.05) and Sphericity Assumption d values were considered to report the data. The comparison of the measurements was examined using mixed-factorial analysis of variance (2 × 4 mixed ANOVA) to determine if the MD-1, MDpre, MDpost and MD+3 measurements (Time) in the player status (Group: starter and non-starter) showed a significant level of difference based on group and time. In cases of significant difference, pairwise comparisons were analyzed with the Bonferroni test. To demonstrate the power of the statistical analysis, effect size values using Eta squared were utilized (partial eta squared (ηp^2^) indicates the proportion of variance in the dependent variable that is explained by a specific independent variable). ηp^2^ values less than 0.25, between 0.26 and 0.63, and greater than 0.63 were classified as small, medium, and large effect sizes, respectively (Richardson, 2011). Moreover, Cohen’s d effect size (ES) with 95% confidence interval was calculated to define the magnitude of pairwise differences. The ES magnitude was defined as follows: <0.2 = trivial, 0.2 to 0.6 = small effect, >0.6 to 1.2 = moderate effect, >1.2 to 2.0 = large effect, and >2.0 = very large (Hopkins et al., 2009). Subsequently, multiple mixed ANOVA were employed for obtaining differences between s-IgA and s-Cort in starting and non-starting players during successful matches (won) and unsuccessful matches (drawn or lost). Indeed, the same mixed ANOVA were performed for observing differences between s-IgA and s-Cort in MD-1, MDpre, MDpost and MD+3 in soccer players starters and non-starters in successful matches and unsuccessful matches. A Pearson correlation coefficient r was used to examine the relationship between starters and non-starters players considering the moment of the week (MD-1 and MDpre) and (MDpre, MDpost and MD+3) for successful and unsuccessful matches. To interpret the magnitude of these correlations, the following criteria was adopted: r ≤ 0.1, trivial; 0.1 < r ≤ 0.3, small; 0.3 < r ≤ 0.5, moderate; 0.5 < r ≤ 0.7, large; 0.7 < r ≤ 0.9, very large; and r > 0.9, almost perfect.

Finally, a multiple regression analysis was used to model the prediction of s-IgA and s-Cort values in starting and non-starting players (MD-1 and MDpre) and remaining values of week (MDpre, MDpost and MD+3). In this regression analysis, all variables were examined separately.

For all statistical analysis, the significance level was set at 5% (*p* < 0.05). Statistical analyses were performed using IBM SPSS Statistics for Windows (version 27.0, IBM Corp Armonk, NY, United States).

## Results

**FIGURE 1:**
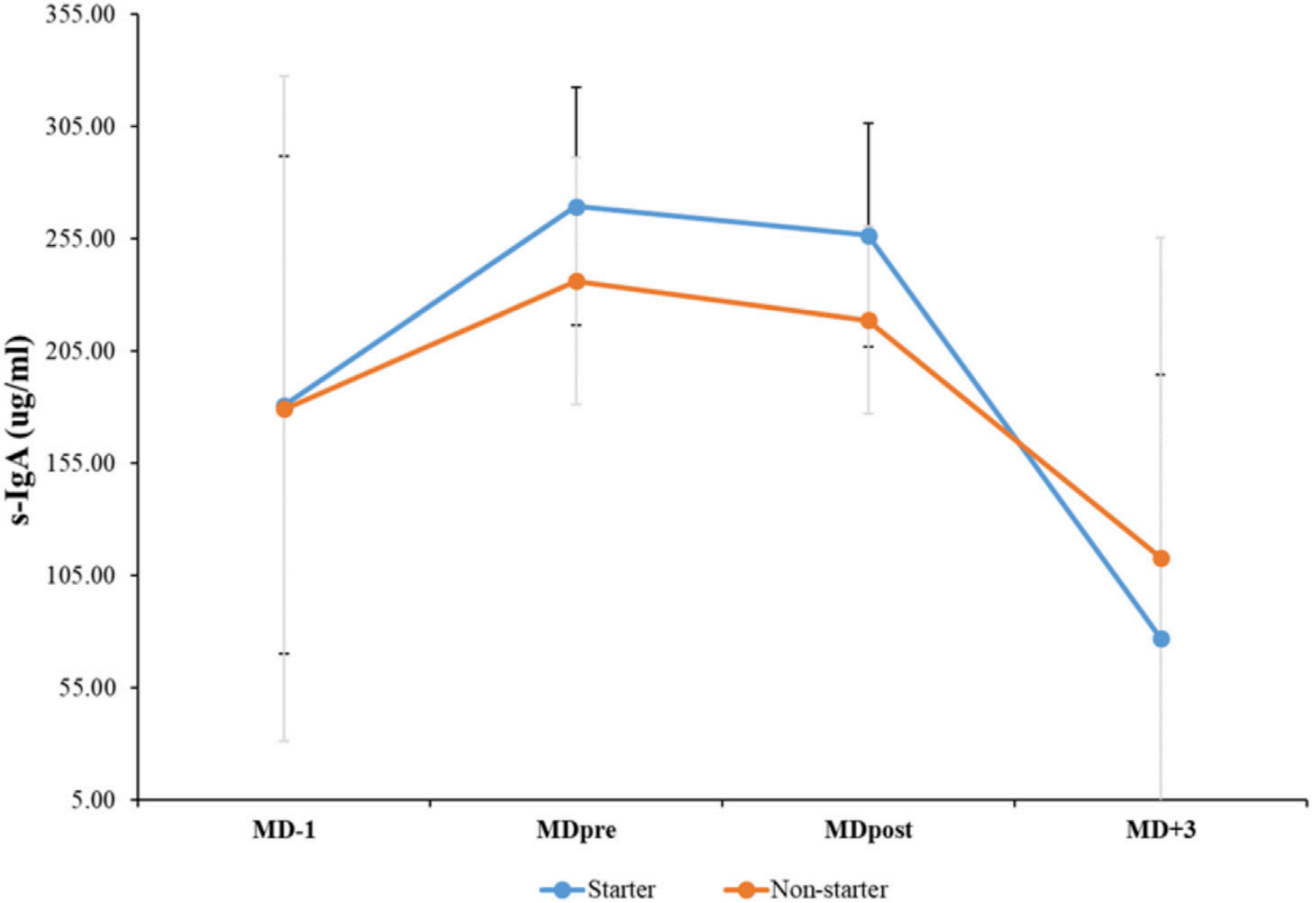
Mean and standard deviation values for salivary immunoglobulin A (s-IgA) at before each match (MD-1), 60-min before kick-off (MDpre), 30-min post-match (MDpost), and 72-h post-match (MD+3).

Figure 1 shows the changes in the mean and standard deviation values for s-IgA at four different time points (MD-1, MDpre, MDpost and MD+3). There was a statistically significant main effect of time for s-IgA values (*p* < 0.001*, F _(3,48)_ = 10.247, ηp^2^ = 0.390, medium effect). According to Bonferroni test, the mean s-IgA value was found to be significantly lower at MD+3 than at MDpre, with an estimated difference of 157.81 μg/mL (t = 4.885, *p* < 0.001*, d = 1.15 (0.5–1.7 95% CI, moderate effect). Moreover, a significant decrease in the mean s-IgA value was observed at MD+3 compared to MDpost, with an estimated difference of 142.61 μg/mL (t = 4.126, *p* < 0.001*, d = 0.97 (0.3–1.5 95% CI, moderate effect)). There were no significant differences observed between starters and non-starters at any time point (*p* = 0.728, F _(1,16)_ = 0.125, ηp^2^ = 0.08, small effect). Lastly, the group-by-time interaction had no significant effect on s-IgA (*p* = 0.630, F _(1,16)_ = 0.581, ηp^2^ = 0.03, small effect).

**FIGURE 2:**
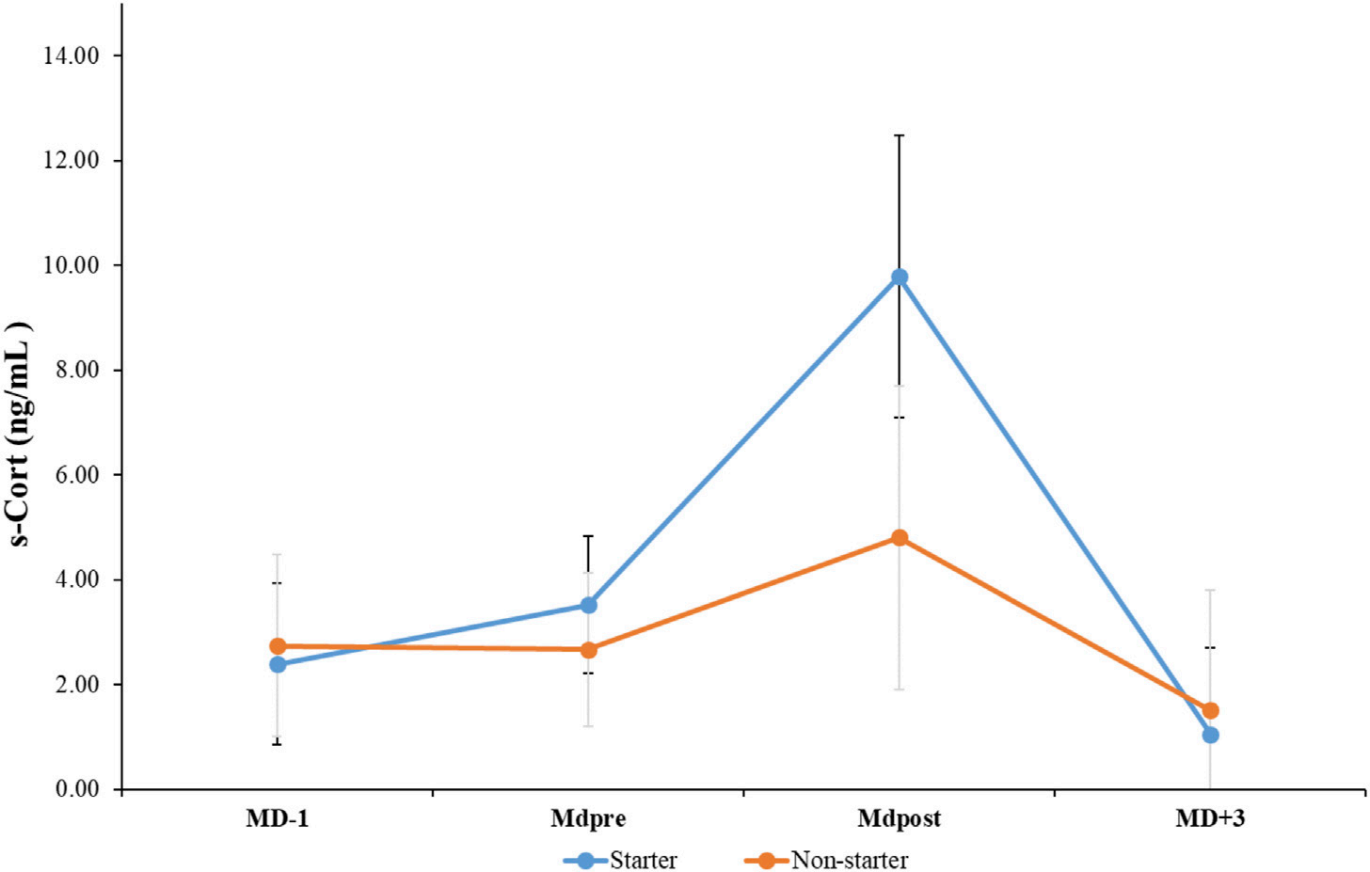
Mean and standard deviation values for cortisol (s-Cort) at before each match (MD-1), 60-min before kick-off (MDpre), 30-min post-match (MDpost), and 72-h post-match (MD+3).

The mean and standard deviation values for s-Cort at the four analyzed time points are presented in Figure 2. A statistically significant main effect of time was observed for s-Cort (*p* < 0.001*, F _(3,48)_ = 28.621, ηp^2^ = 0.641, large effect). The Bonferroni test indicated that s-Cort was significantly higher at MDpost compared to MD-1, with an estimated difference of 4.72 ng/mL (t = 4.256, *p* < 0.001*, d = 1.00 (0.4–1.5 95% CI, moderate effect)). Also, s-Cort values increased significantly at MDpost compared to MDpre, with an estimated difference of 4.19 ng/mL (t = 5.004, *p* < 0.001*, d = 1.17 (0.5–1.7 95% CI, moderate effect)). When compared to MDpre, a statistically significant decrease in s-Cort was observed at MD+3, with an estimated difference of 1.81 ng/mL (t = 4.415, *p* < 0.001*, d = 1.0 (0.4–1.6 95% CI, moderate effect)). Moreover, s-Cort exhibited a statistically significant decline at MD+3 compared to MDpost, with an estimated difference of 6.01 ng/mL (t = 5.401, *p* < 0.001*, d = 1.2 (0.6–1.8 95% CI, large effect)). Additionally, it was found that group effect had an impact on s-Cort mean value, with an estimated difference of 1.25 ng/mL (*p* < 0.05*, F _(1,16)_ = 7.622, ηp^2^ = 0.323, medium effect). Starters displayed higher s-Cort values compared to non-starters during the nine matches (t = 2.761, *p* < 0.05*, d = 0.65 (0.2–1.1 95% CI, moderate effect)). Lastly, there was a significant group-by-time interaction for s-Cort (*p* < 0.01*, F _(3,48)_ = 6.865, ηp^2^ = 0.300, medium effect). The results of the Bonferroni test show that there was a significant increase in s-Cort levels at MDpost compared to MD-1 in starting players, with an estimated difference of 7.38 ng/mL (t = 6.001, *p* < 0.001*, d = 2.0 (0.8–3.1 95% CI, large effect)). There was a significant increase in s-Cort levels in starting players from MDpre to MDpost, with an estimated difference of 6.25 ng/mL (t = 6.163, *p* < 0.001*, d = 2.05 (0.8–3.2 95% CI, large effect)). For MDpost, it was found that starters had significantly higher s-Cort values compared to non-starters, with an estimated difference of 4.97 ng/mL (t = 3.771, *p* < 0.01*, d = 1.77 (0.6–2.8 95% CI, moderate effect)). There was also a significant decrease in s-Cort values in starters from MDpost to MD+3, with an estimated difference of 8.72 ng/mL (t = 6.636, *p* < 0.001*, d = 2.21 (0.9–3.4 95% CI, large effect)). Lastly, it was observed that there was a statistically significant decrease in s-Cort levels in non-starting players compared to MDpost at MD+3, with an estimated difference of 3.29 ng/mL (t = 2.534, *p* < 0.05*, d = 0.84 (0.0–1.5 95% CI, moderate effect)).

A mixed ANOVA with s-IgA values of starting and non-starting players from successful and unsuccessful matches did not reveal a significant difference between groups (Table 1). Pairwise comparisons revealed significant differences in MDpre, MDpost and MD+3 (*p* < 0.05). However, similar analysis with s-Cort values of starting and non-starting players in matches won, showed a significant difference between starters and non-starters, F (1.53) = 5.97, *p* = 0.02, η2 = 0.10. The dataset also revealed higher values in non-starters than starters. Furthermore, s-Cort in starters and non-starters from unsuccessful matches did not show a significant difference between groups (See Table 1). Finally, pairwise comparisons showed significant differences at MDpre (*p* = 0.02).

**TABLE 1:** Mean values of s-IgA and s-Cort and a mixed ANOVA between starting and non-starting players following successful and unsuccessful matches.

		Successful matches									
		s-IgA					s-Cort				
	Starters	Non-starters		*F*	*p*	η^2^_partial_	Starters	Non-starters	*F*	*p*	η^2^_partial_
MD-1	205.73 ± 123.98	163.30 ± 93.81		1.92	0.86	0.06	6.19 ± 3.52	5.85 ± 7.31	0.05	0.82	0.00
	CV: 60.26	CV: 57.44					CV: 58.88	CV: 124.86			
MDpre	132.15 ± 85.39	169.30 ± 143.01		1.48	0.23	0.03	4.80 ± 2.10	3.46 ± 1.90	5.97	0.02*	0.10
	CV: 64.61	CV: 84.46					CV: 43.74	CV: 54.89			
MDpost	146.27 ± 79.46	181.09 ± 106.90		1.92	0.17	0.03	9.70 ± 5.94	11.81 ± 8.06	1.26	0.27	0.02
	CV: 54.32	CV: 59.03					CV: 61.22	CV: 67.19			
MD+3	167.68 ± 136.87	152.90 ± 77.15		<1.00	0.65	0.00	6.55 ± 3.80	7.05 ± 6.60	<1.00	0.72	0.00
	CV: 81.62	CV: 50.45					CV: 58.01	CV: 93.56			
**Unsuccessful matches**											
MD-1	213.41 ± 125.27		179.39 ± 90.95	1.19	0.28	0.02	4.88 ± 3.38	5.01 ± 3.81	<1.00	0.90	0.00
	CV: 58.69		CV: 50.70				CV: 69.19	CV: 76.06			
MDpre	218.76 ± 149.30		149.52 ± 72.87	3.84	0.06	0.07	4.68 ± 3.49	3.97 ± 2.11	<1.00	0.41	0.01
	CV: 68.25		CV: 48.73				CV: 74.69	CV: 53.24			
MDpost	239.31 ± 120.79		177.81 ± 97.88	3.68	0.06	0.07	10.46 ± 4.90	11.60 ± 7.17	<1.00	0.51	0.01
	CV: 50.47		CV: 55.04				CV: 46.92	CV: 61.85			
MD+3	237.55 ± 172.82		209.70 ± 137.62	<1.00	0.51	0.01	4.85 ± 2.72	6.61 ± 4.46	3.19	0.08	0.06
	CV: 72.75		CV: 65.62				CV: 56.12	CV: 67.60			

Note: ∗ denotes significance at *p* < 0.05, and ∗∗ denotes significance at *p* < 0.01. Coefficient of Variation (CV); Before each match (MD-1), 60-min before kick-off (MDpre), 30-min post-match (MDpost), and 72-h post-match (MD+3). Salivary immunoglobulin A (s-IgA) and cortisol (s-Cort).

A mixed ANOVA with s-IgA values between MD-1, MDpre, MDpost and MD+3 in starters and non-starters from successful and unsuccessful matches revealed a significant difference in MDpre and MDpost for starters, F (1.53) = 8,10, *p* = 0.01, η2 = 0.12, F (1.53) = 13,13, *p* = 0.001, η2 = 0.18, respectively. However, the data did not reveal any significant difference in the other values or for the non-starting players. In this context, pairwise comparisons showed significant differences at MD-1, and MD+3 (*p* < 0.02) (See Table 2). Furthermore, a new mixed ANOVA with s-Cort values between MD-1, MDpre, MDpost and MD+3 in starters and non-starters in successful and unsuccessful matches revealed a significant difference at MD+3 in starting players, F (1.53) = 3.96, *p* = 0.05, η2 = 0.06. Nevertheless, the data did not reveal any significant difference in any of the other values or in the non-starting players. Lastly, pairwise comparisons revealed significant differences at MD-1, MDpre, MDpost (*p* < 0.01) (See Table 2).

**TABLE 2:** Mean values of s-IgA and s-Cort and a mixed ANOVA between MD-1, MDpre, MDpost and MD+3 in starting and non-starting players following successful and unsuccessful matches.

		s-IgA				s-Cort				
	Starters in successful matches	Starters in unsuccessful matches	*F*	*p*	η^2^_partial_	Starters in successful matches	Starters in unsuccessful matches	*F*	*p*	η^2^_partial_
MD-1	205.73 ± 123.98	213.41 ± 125.27	<1.00	0.81	0.00	6.19 ± 3.52	5.85 ± 7.31	2.20	0.14	0.04
	CV: 60.26	CV: 58.69				CV: 58.88	CV: 124.86			
MDpre	132.15 ± 85.39	218.76 ± 149.30	8.10	0.01*	0.12	4.80 ± 2.10	3.46 ± 1.90	<1.00	0.86	0.00
	CV: 64.61	CV: 68.25				CV: 43.74	CV: 54.89			
MDpost	146.27 ± 79.46	239.31 ± 120.79	13.13	0.001**	0.18	9.70 ± 5.94	11.81 ± 8.06	<1.00	0.59	0.00
	CV: 54.32	CV: 50.47				CV: 61.22	CV: 67.19			
MD+3	167.68 ± 136.87	237.55 ± 172.82	3.15	0.08	0.05	6.55 ± 3.80	7.05 ± 6.60	3.96	0.05*	0.06
	CV: 81.62	CV: 72.75				CV: 58.01	CV: 93.56			
	Non-Starters successful in matches	Non-Starters in unsuccessful matches	*F*	*p*	η^2^_partial_	Non-starters in successful matches	Non-starters in unsuccessful matches	*F*	*p*	η^2^_partial_
MD-1	163.30 ± 93.81	179.39 ± 90.95	<1.00	0.56	0.01	5.85 ± 7.31	5.01 ± 3.81	<1.00	0.63	0.01
	CV: 57.44	CV: 50.70				CV: 124.86	CV: 76.06			
MDpre	169.30 ± 143.01	149.52 ± 72.87	<1.00	0.57	0.01	3.46 ± 1.90	3.97 ± 2.11	<1.00	0.70	0.02
	CV: 84.46	CV: 48.73				CV: 54.89	CV: 53.24			
MDpost	181.09 ± 106.90	177.81 ± 97.88	<1.00	0.92	0.00	11.81 ± 8.06	11.60 ± 7.17	<1.00	0.93	0.00
	CV: 59.03	CV: 55.04				CV: 67.19	CV: 61.85			
MD+3	152.90 ± 77.15	209.70 ± 137.62	2.72	0.11	0.06	7.05 ± 6.60	6.61 ± 4.46	<1.00	0.95	0.00
	CV: 50.45	CV: 65.62				CV: 93.56	CV: 67.60			

Note: ∗ denotes significance at *p* < 0.05, and ∗∗ denotes significance at *p* < 0.01. Coefficient of Variation (CV); Before each match (MD-1), 60-min before kick-off (MDpre), 30-min post-match (MDpost), and 72-h post-match (MD+3). Salivary immunoglobulin A (s-IgA) and cortisol (s-Cort).

Furthermore, a different correlation analysis were performed between starters and non-starters considering MD-1 and MDpre and MDpre, MDpost and MD+3 for matches won, drawn and lost (See Table 3).

**TABLE 3:** Correlation analysis between starters and non-starters considering MD-1 and MDpre and MDpre, MDpost and MD+3 for successful and unsuccessful matches.

		Successful matches						
		MDpre	MDpost	MD+3		MDpre	MDpost	MD+3
	s-IgA							
Starters	MD-1	*r* = 0.62	*r* = 0.74	*r* = 0.85	Non-Starters	*r* = 0.65	*r* = 0.57	*r* = 0.71
		*p* = 0.001**	*p* = 0.001**	*p* = 0.001**		*p* = 0.001**	*p* = 0.01*	*p* = 0.001**
	MDpre		*r* = 0.50	*r* = 0.56			*r* = 0.88	*r* = 0.54
			*p* = 0.001**	*p* = 0.001**			*p* = 0.001**	*p* = 0.01*
	**s-Cort**							
Starters	MD-1	*r* = −0.09	*r =* -0.05	*r* = 0.27	Non-Starters	*r* = −0.17	*r =* -0.16	*r* = 0.00
		*p* = 0.63	*p* = 0.80	*p* = 0.13		*p* = 0.45	*p* = 0.46	*p* > 0.99
	MDpre		*r* = −0.04	*r* = 0.01			*r* = 0.14	*r* = 0.11
			*p* = 0.84	*p* = 0.95			*p* = 0.54	*p* = 0.61
		**Unsuccessful Matches**						
	**s-IgA**							
Starters	MD-1	*r* = 0.66	*r* = 0.47, *p* = 0.01*	*r* = 0.64, *p* = 0.001**	Non-Starters	*r* = −0.21, *p* = 0.35	*r* = 0.15	*r* = 0.02
		*p* = 0.001**					*p* = 0.52	*p* = 0.92
	MDpre		*r* = 0.70, *p* = 0.001**	*r* = 0.74, *p* = 0.001**			*r* = 0.39	*r* = 0.15
							*p* = 0.08	*p* = 0.52
	**s-Cort**							
Starters	MD-1	*r* = 0.12	*r =* 0.20	*r* = 0.35	Non-Starters	*r* = 0.65	*r =* -0.33	*r* = 0.01
		*p* = 0.55	*p* = 0.29	*p* = 0.07		*p* = 0.001**	*p* = 0.14	*p* = 0.98
	MDpre		*r* = 0.17	*r* = 0.06			*r* = −0.12	*r* = 0.00
			*p* = 0.39	*p* = 0.77			*p* = 0.61	*p* = 0.99

Note: ∗ denotes significance at *p* < 0.05, and ∗∗ denotes significance at *p* < 0.01. Before each match (MD-1), 60-min before kick-off (MDpre), 30-min post-match (MDpost), and 72-h post-match (MD+3). Salivary immunoglobulin A (s-IgA) and cortisol (s-Cort).

Finally, a multiple regression analysis was performed on starting and non-starting players for successful and unsuccessful matches to verify which moment during the week (agreement with the correlation analysis) may be used to further explain the importance of s-IgA and s-Cort responses (See Table 4).

**TABLE 4:** Values of regression analysis explaining the association between variables.

			Successful matches					
	s-IgA							
			R	*R* ^2^	Adjusted *R* ^2^	*F*	*p*	SE
Starters	MD-1	MDpre	0.62	0.39	0.37	19.69	0.001**	67.85
		MDpost	0.74	0.55	0.53	37.18	0.001**	54.43
		MD+3	0.85	0.63	0.51	80.68	0.001**	73.27
	MDpre	MDpost	0.50	0.25	0.22	10.18	0.001**	70.04
		MD+3	0.56	0.22	0.20	14.34	0.001**	114.99
Non-Starters	MD-1	MDpre	0.65	0.42	0.39	13.73	0.001**	114.92
		MDpost	0.57	0.32	0.29	9.46	0.01*	90.26
	MDpre	MD+3	0.71	0.50	0.47	18.90	0.001**	56.04
		MDpost	0.88	0.77	0.76	64.61	0.001**	52.93
		MD+3	0.54	0.29	0.25	7.77	0.01*	66.68
			**Unsuccessful Matches**					
Starters	MD-1	MDpre	0.66	0.44	0.42	21.35	0.001**	113.62
		MDpost	0.47	0.22	0.20	7.83	0.01*	108.30
		MD+3	0.64	0.41	0.39	19.01	0.001**	134.82
	MDpre	MDpost	0.70	0.49	0.48	26.34	0.01*	87.52
		MD+3	0.74	0.60	0.47	33.18	0.01*	117.88
	**s-Cort**							
Non-Starters	MD-1	MDpre	0.65	0.43	0.40	14.06	0.001**	1.64

Note: ∗ denotes significance at *p* < 0.05, and ∗∗ denotes significance at *p* < 0.01. Before each match (MD-1), 60-min before kick-off (MDpre), 30-min post-match (MDpost), and 72-h post-match (MD+3). Salivary immunoglobulin A (s-IgA) and cortisol (s-Cort).

## Discussion

The main results of the present study revealed that the mean s-IgA value was found to be significantly lower at MD+3 compared to MDpre and MDpost, while s-Cort was significantly higher at MDpost compared to both MD-1 and MDpre. Furthermore, a statistically significant decrease in s-Cort was observed at MD+3 compared to both MDpre and MDpost. There was also a significant group-by-time interaction for s-Cort. There was a significant increase in s-Cort levels at MDpost compared to MD-1 and from MDpre to MDpost in starting players. For MDpost, it was found that starters had significantly higher s-Cort values compared to non-starters. The s-IgA values of starting and non-starting players from successful and unsuccessful matches did not reveal a significant difference between groups. However, similar analysis of s-Cort in matches won, showed a significant difference between starters and non-starters. s-IgA values between MD-1, MDpre, MDpost and MD+3 in starters and non-starters from successful and unsuccessful matches revealed significant difference in MDpre and MDpost in starters, respectively. The s-Cort values between MD-1, MDpre, MDpost and MD+3 in starters and non-starters in successful and unsuccessful matches revealed significant differences at MD+3 in starting players.

One of the main findings of the present study was the significantly lower s-IgA concentration at MD+3 (following a post-match recovery period) compared to both MDpre and MDpost time-points. However, when considering playing status, starters and non-starters revealed higher values at MD+3 than MDpost (successful matches). No difference between pre- and post-match or between MD-1 and the other time-points was observed. Moreover, the player’s status (starters vs. non-starters groups) did not influence s-IgA responses, as no significant differences were observed between these groups at any time point. This may suggest that an accumulated effect from playing successive official matches may have occurred. These findings are aligned with data from previous studies in soccer players that demonstrated the impact of psychophysiological stress on the mucosal immune function, with decreases in s-IgA concentration across periods of congested fixtures or intensive training loads in both professional and elite youth soccer players (Morgans et al., 2014; Mortatti et al., 2012; Mortatti et al., 2020; Moreira et al., 2016). Additionally, no significant differences were found between starters and non-starters regardless of match outcome (successful *versus* unsuccessful), although unsuccessful matches reported higher values for starters than non-starters at MDpre and MDpost. Such findings may suggest that unsuccessful matches had an influence on starting players.

When the mean data from the four time points for the nine assessed matches was considered, this response (accumulated effect) may partly provide a reasonable explanation for the findings rather than only the impact of an acute stress (i.e., from pre-to post-match). For example, Owen et al. (2016) reported a decrease in s-IgA during a 7-day period of intensified soccer training, and Mortatti et al. (2012) showed a reduction in s-IgA concentration together with an increase in the incidence of URTI in elite U-19 soccer players monitored in a series of seven matches in 20 days. Moreover, Moreira et al. (2016) also reported a decrease in s-IgA concentrations in elite youth soccer players from the first saliva collection time point compared with the last, during a congested match schedule (seven matches played in 7 days). The findings of this study (Moreira et al., 2016) demonstrated that accumulated fatigue related to participation in a congested match schedule may negatively affect the mucosal immunity of soccer players. Indeed, Morgans et al. (2014) revealed fluctuations in s-IgA related to changes in the physical match demands placed on soccer players during congested fixture schedules. The authors reported a decrease in s-IgA during these periods (2-3 matches per week) but did not observe these responses when a normal fixture schedule returned (one match per week). Our data adds to the existing knowledge that the decrease in s-IgA concentration (following 48-h post-match) may occur irrespective of starting status (i.e., starters vs. non-starters). Our hypothesis that differing s-IgA responses may be verified when considering the starting status of players was therefore refuted. However, when considering successful and unsuccessful match outcome, those findings were not consistent. For instance, s-IgA increased at MD+3 for starters after a successful match outcome (win). However, when considering an unsuccessful match outcome (draw or loss), s-IgA remained similar at MD+3 for starters and increased for non-starters.

Notably in a recent publication with the same elite professional male soccer players (Morgans et al., 2022) as in the present study, it was observed that when performing a higher workload, players tend to present a slower return to initial s-IgA concentration. Longer playing time, greater total distance covered, higher number of high-intensity accelerations, and greater high-intensity distance covered involved s-IgA differences between 48-h post-match and other assessed time-points. Interestingly, Springham et al. (2022) recently reported no training and match load variables related to s-IgA changes in elite-level professional male soccer players from one English Championship team across a competitive season. While the present study examined previously published data (Morgans et al., 2022) from the same cohort, it may add novel and unique information to the current literature, in particular for elite professional soccer players, as the results of the present study may be, at least in part, explained by the well-known involvement of lifestyle factors, psychological stress, and physiological factors in regulating s-IgA. Although more research is needed regarding the influence of match outcome.

In relation to s-Cort, there was a significant difference between MD-1 and MDpost and, also between MDpre and MDpost. An increase in s-Cort was observed from both MD-1 and MDpre to MDpost indicating a characteristic response of increased activation of the hypothalamic-pituitary-adrenal (HPA) axis due to a psychophysiological stress related to participating in official match-play (Venkataraman et al., 2007). While exercise *per se* (physical stress), may represent a potent physiological stressor, where elevations in s-Cort levels have been found to enhance cardiovascular activity, raise glucose levels, and promote anti-inflammatory responses, it has been demonstrated that acute psychological stressors have a significant impact on cortisol responses (Bishop and Gleeson, 2009). Soccer match-play as a sports competition, is a typical model of an uncontrollable and social-evaluative task (Dickerson and Kemeny, 2004), and that the pressure of participating in official matches, may be considered as additional and very important stress factor to the well-known physiological role of cortisol during exercise. This situation may lead to a greater level of player stress resulting in a higher s-Cort response which is supported by the results of the present study. The current study revealed that starters presented significantly higher s-Cort values at MDpre than non-starters prior to successful matches. Furthermore, similarly, following unsuccessful matches, although without any significant results, a significantly lower value was observed at MD+3 for starters compared to non-starters.

Moreira et al. (2009) observed an increasing trend in s-Cort with no statistical significance following a competitive training match in top-level male professional soccer players divided into two teams. The authors suggested that the absence of the expected increases in s-Cort in some players may be explained, at least in part, by situational stress related to official competition and individual aspects such as coping mechanisms. The authors summarized that when the competition element is removed, the related match stress does not appear strong enough to induce a significant impact on endocrinal responses. The results of this study were aligned with those reported earlier by Haneishi et al. (2007), who found increased s-Cort values post-match for both starters and non-starters of female National Collegiate Athletic Association Division I collegiate soccer players, after one regular match, but not for one typical practice session. The previous findings are in contrast with the results of the present study as a significantly lower value was observed at MD+3 for starters when compared to non-starters. Similarly, a significantly higher value was found at MD+3 for starters following successful match outcome when compared with unsuccessful match outcomes.

Furthermore, the earlier results from Moreira et al. (2009) were more recently corroborated by Jiménez et al. (2020) who demonstrated that the importance (seriousness) and competition level of performance may modulate s-Cort responses (and also salivary testosterone responses). In conjunction, among others, these data suggest that physiological, psychological, and situational variables combined may modulate the HPA responses during official match-play in soccer players. Recently, Morgans et al. (2022) demonstrated that s-Cort increased from 60-min prior to kick-off to 30-min post-match in male professional soccer players, and that at 48-h post-match, a decrease in s-Cort concentration was observed. Indeed, Pinto et al. (2021) showed similar results during four successive soccer matches in U-17 male soccer players, reporting an increase from pre-to post-match in s-Cort for this population, but with non-significant changes in s-Cort levels across the four assessed matches.

The current data, however, revealed a time-dependent effect in starters, displaying a higher s-Cort concentration compared to non-starters during the nine matches. Additionally, the match outcome seems to impact starters values at MD+3 by revealing higher values following successful rather than unsuccessful match outcomes. In conjunction, these findings suggest that while participating in official match-play may modulate stress responses regardless of the player’s status, starting players would demonstrate an amplified stress response, which may be related to both the psychological factor, higher workload demand during the previous week and the match outcome, namely, won matches. The s-Cort levels in the body have been identified as a significant factor in preparing the body for physical activity, as well as serving as a reliable biomarker for psychosocial stress (Venkataraman et al., 2007; Bishop and Gleeson, 2009).

Another important finding of the present study that deserves to be highlighted is the fact that despite the increase in s-Cort from pre-to post-match, and a reduction in s-Cort at MD+3, no differences in s-IgA from pre-to post-match were observed, and inversely, a reduction in s-IgA concentration was identified at MD+3. These results do not support a short-term inhibitory effect of increased cortisol concentration on transcytosis (transport of IgA across the epithelial membrane into the salivary ducts) (Bishop and Gleeson, 2009). On the other hand, this dissociation between s-Cort and s-IgA concentration responses has been reported for two final matches played 3 days apart by professional female soccer players (Maya et al., 2016). Maya et al. (2016) showed that s-Cort increased only after the first final match (from pre-to post-match = +116%), while s-IgA concentration did not change following any examined match. Moreover, Moreira et al. (2016) observed no changes in s-Cort concentration across match time points (congested match schedule; seven matches played in 7 days), but, in contrast, reported a decrease in s-IgA concentration. However, as a unique finding of the present study, due to the study design, it was demonstrated that despite no changes in s-IgA from pre-to post-match, regardless of the increases in s-Cort concentration, a significant reduction in s-IgA was observed at MD+3, despite the reduction in s-Cort at this time-point. The present finding not only corroborates other research demonstrating that s-Cort does not play a role in the regulation of s-IgA secretion but shows that it is imperative to monitor this biomarker across the season considering both the acute responses to match-play, and changes from match to match. However, a different scenario occurred between starters and non-starters when match outcome was considered. For instance, s-IgA increased at MD+3 for starters following a successful match outcome (win). However, when considering unsuccessful matches, s-IgA remained similar at MD+3 for starters although increased for non-starters.

Notwithstanding, the regression analysis presented for successful and unsuccessful matches showed that MD-1 and MDpre may partly explain the results of the data collection independent of the match outcome or playing status. Such findings reinforce more research is warranted to confirm the present findings, since to the authors best knowledge, this is the first study to test such a hypothesis. Moreover, monitoring the s-Cort and s-IgA concentration at MD+3, would allow coaches and staff members to improve their training plan to avoid increasing training exposure when some players would still be presenting a higher risk for URTI given a reduction in mucosal immunity function, or even, when a persistent elevated s-Cort levels is observed. Considering the player’s playing status would also support the optimization and individualization of the training and recovery process.

### Limitations and future directions

The present study contains some limitations that should be noted. The sample size from only one team represents a real-world soccer environment, although is a limitation of the present study. Only a 10-week period with nine official matches was analyzed and thus, full-season and different periods of pre-season and in-season could strengthen the research as recently suggested (Morgans et al., 2022). In this regard, it would also be possible to consider congested periods *versus* non-congested (Morgans et al., 2014b; Moreira et al., 2016) and/or decisive matches (i.e., elimination matches) as previously suggested (Maya et al., 2016). Finally, when dividing the data into successful *versus* unsuccessful matches, the number of matches is lower which should be considered in future studies.

Future research should include the analysis of URTI and identify any relationship with s-IgA, as previously it was shown that in other sports there was an inverse correlation (Rico-Gonzalez et al., 2021), although in soccer such an assertion was not confirmed (Rico-Gonzalez et al., 2021b). Thus, it may be suggested to monitor low levels of s-IgA to avoid mucosal immune system suppression. Finally, a further suggestion may be the inclusion of other wellness and fatigue variables as previously highlighted (Morgans et al., 2022).

## Conclusion

The present findings confirmed the study hypothesis, and it seems that both playing status and match outcome influence s-IgA and s-Cort responses of elite soccer players. Additionally, the current results do not support a short-term inhibitory effect of increased s-Cort concentration on s-IgA responses. The findings demonstrate that despite the rise in s-Cort concentration from pre-to-post match, no changes in s-IgA were observed during these time-points. On the other hand, a significant reduction in s-IgA was noted at MD+3, despite the decrease in s-Cort at this time-point. This suggests a compromised function of the main mucosal immune function marker that is not aligned with s-Cort concentration.

This unique data implies that practitioners should be mindful of the vulnerability of soccer players 48 h post-match, increasing the risks of URTI and illness in general, although this was not controlled in the present research. However, staff should also include the load differences between starters and non-starters and the match outcome. For starters, s-IgA increased at MD+3 following a successful match outcome (win). Conversely, in unsuccessful matches, s-IgA remained similar at MD+3 for starters, although it increased for non-starters. These situational factors should be taken into account in monitoring assessments in practical real-world settings.

In summary, the present study suggests that in elite level soccer players, there is a need for a more comprehensive monitoring system that may include regular measurements of s-IgA and s-Cort in conjunction with workload data and behavioural metrics to detect the influence of these components on changes in s-IgA and s-Cort. Consequently, to improve practitioners’ understanding of individual changes in the psychophysiological status of soccer players, allowing a more individualized approach to training.

## Data Availability

The raw data supporting the conclusions of this article will be made available by the authors, without undue reservation.
